# Supplementary material for: Causal Relationship between Aging and Anorexia Nervosa: A White-Matter-Microstructure-Mediated Mendelian Randomization Analysis
Source: Biomedicines. 2024 Aug 16;12(8):1874. doi: 10.3390/biomedicines12081874 (PMC11351342; doi:10.3390/biomedicines12081874)
Supplement: Supplementary file 1 [file biomedicines-12-01874-s001.zip › Supplementary Figure/Figure S4/Singe SNP plot of MR analysis for TL to ubm-b-2002.pdf]

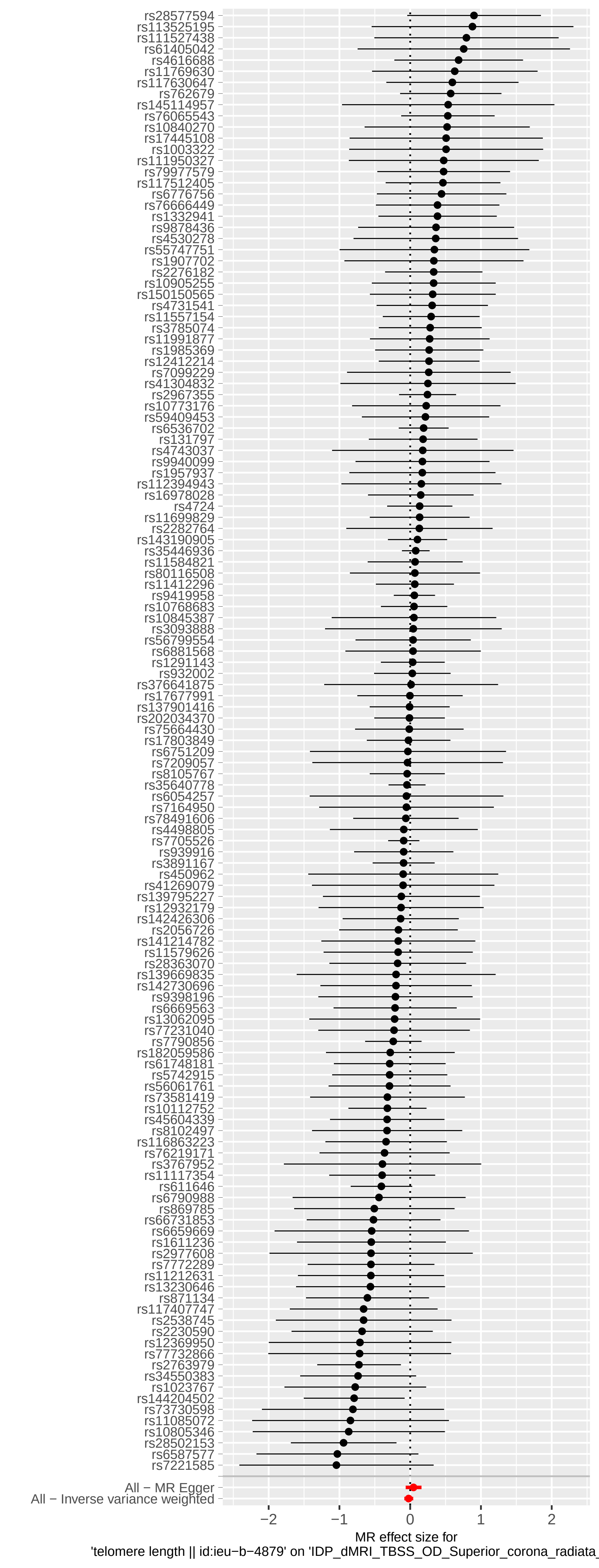
rs28577594
rs113525195
rs111527438
rs61405042
rs4616688
rs11769630
rs117630647
rs762679
rs145114957
rs76065543
rs10840270
rs17445108
rs1003322
rs111950327
rs79977579
rs117512405
rs6776756
rs76666449
rs1332941
rs9878436
rs4530278
rs55747751
rs1907702
rs2276182
rs10905255
rs150150565
rs4731541
rs11557154
rs3785074
rs11991877
rs1985369
rs12412214
rs7099229
rs41304832
rs2967355
rs10773176
rs59409453
rs6536702
rs131797
rs4743037
rs9940099
rs1957937
rs112394943
rs16978028
rs4724
rs11699829
rs2282764
rs143190905
rs35446936
rs11584821
rs80116508
rs11412296
rs9419958
rs10768683
rs10845387
rs3093888
rs56799554
rs6881568
rs1291143
rs932002
rs376641875
rs17677991
rs137901416
rs202034370
rs75664430
rs17803849
rs6751209
rs7209057
rs8105767
rs35640778
rs6054257
rs7164950
rs78491606
rs4498805
rs7705526
rs939916
rs3891167
rs450962
rs41269079
rs139795227
rs12932179
rs142426306
rs2056726
rs141214782
rs11579626
rs28363070
rs139669835
rs142730696
rs9398196
rs6669563
rs13062095
rs77231040
rs7790856
rs182059586
rs61748181
rs5742915
rs56061761
rs73581419
rs10112752
rs45604339
rs8102497
rs116863223
rs76219171
rs3767952
rs11117354
rs611646
rs6790988
rs869785
rs66731853
rs6659669
rs1611236
rs2977608
rs7772289
rs11212631
rs13230646
rs871134
rs117407747
rs2538745
rs2230590
rs12369950
rs77732866
rs2763979
rs34550383
rs1023767
rs144204502
rs73730598
rs11085072
rs10805346
rs28502153
rs6587577
rs7221585
All − MR Egger
All − Inverse variance weighted
−2
−1
0
1
2
MR effect size for
'telomere length || id:ieu−b−4879' on 'IDP_dMRI_TBSS_OD_Superior_corona_radiata_
